# Clinical Trial on the Usefulness of On-Site Evaluation of Canine Fetal Fluids by Reagent Test Strip in Puppies at Elective Caesarean Section

**DOI:** 10.3390/biology11010038

**Published:** 2021-12-27

**Authors:** Jasmine Fusi, Barbara Bolis, Monica Probo, Massimo Faustini, Augusto Carluccio, Maria Cristina Veronesi

**Affiliations:** 1 Department of Veterinary Medicine, Università degli Studi di Milano; Via dell’Università 6, 26900 Lodi, Italy; jasmine.fusi@unimi.it (J.F.); barbarabolis.medicoveterinario@gmail.com (B.B.); Massimo.faustini@unimi.it (M.F.); maria.veronesi@unimi.it (M.C.V.); 2 Faculty of Veterinary Medicine, Veterinary Teaching Hospital University of Teramo, Località Piano D’Accio, 64100 Teramo, Italy; acarluccio@unite.it

**Keywords:** dog, newborn, fetal fluid composition, prompt assistance

## Abstract

**Simple Summary:**

In veterinary medicine, the availability of an easy tool for the immediate evaluation of biological fluids’ composition on site is desirable and beneficial as a first-line diagnostic instrument in many instances and in several animal species. The aim of the present study was to assess the usefulness of reagent urinary test strips marketed for urines for the on-field evaluation of fetal fluids’ composition in newborn dogs at elective caesarean section. From surviving newborns, a collection of 113 amniotic and 107 allantoic samples was performed, and 8 amniotic and allantoic fluids were collected from non-surviving newborns. All the fetal fluids were assessed by urinary test strips within 5 min from collection. Significant differences were found for both types of fluid between surviving and non-surviving puppies, and between medium/large and small-sized puppies. Differences were also depicted between amniotic and allantoic fluids within the surviving group. These results seem to suggest that the on-site analysis of fetal fluids’ composition by reagent test strips could represent a first-line tool for the evaluation of puppies and for the quick identification of puppies requiring special monitoring and assistance.

**Abstract:**

The reagent urinary test strips (TS) marketed for urines represent the first-line diagnostic tool in many instances. Therefore, the aim of the study was to assess the usefulness of TS for the on-field evaluation of fetal fluids’ composition in newborn dogs at elective caesarean section. Of a total of 137 puppies born at term, 127 survived and 10 did not survive. One hundred and thirteen amniotic and 107 allantoic samples from surviving newborns were collected, and 8 amniotic and allantoic fluids were collected from the non-surviving newborns and assessed by strips. Significantly lower amounts of amniotic glucose and higher amounts of amniotic and allantoic nitrites, amniotic protein, allantoic urobilinogen, and amniotic bilirubin concentrations were found in non-surviving when compared to surviving newborns. In the surviving ones, higher specific gravity and bilirubin concentrations, and lower pH, were found in allantoic than in amniotic fluids. Higher amniotic and allantoic glucose concentrations, higher amniotic and allantoic pH, and lower amniotic and allantoic protein concentrations were found in medium/large- than in small-sized puppies. The TS allowed the quick evaluation of fetal fluids in puppies at birth. The differences between surviving and non-surviving puppies seem to suggest that the on-site analysis of fetal fluids’ composition by TS could represent a first-line diagnostic tool in the field of canine neonatology, allowing the quick recognition of puppies needing assistance as a complementary tool for clinical evaluation.

## 1. Introduction

In mammals, fetal fluids hold a significant role in the process of fetal development and growth and reflect fetal well-being. As reported by Underwood et al. [1], amniotic fluid cannot be considered only as fetal urines, but instead a complex fluid provided by different sources and with a variegate composition. In humans, the amniotic fluid collected through amniocentesis has been demonstrated to be very useful for many investigations, such as chromosomal abnormalities diagnosis, gender definition, infections and/or inflammation diagnosis, microbiota studies, and fetal pulmonary maturity assessment [2,3]. Different to humans, in dogs, as in other animal species, beside the amniotic cavity containing the amniotic fluid, the allantoic sac also persists until term of pregnancy and, because of the communication with the fetal urinary system, it plays a role as a fetal metabolism waste reservoir [4]. Therefore, allantoic fluid accumulates, especially during the second half of pregnancy [5].

The composition and amount of fetal fluid changes widely during gestation, with the final form reflecting not only the simple filtration from maternal blood, but also resulting from the activity of the fetus itself [6]. The characterization of normal canine fetal fluids’ composition deserves interest not only for a better understanding of canine perinatology, but also for the future identification of possible diagnostic markers of fetal/neonatal diseases or well-being for a better management of newborn puppies requiring special assistance after birth [7]. Because of the polytocous characteristics of the dog and of the canine placenta features, the amniocentesis during pregnancy in dogs has been considered difficult and possibly harmful for the fetuses until recently [5]. However, a study [7] showed that it can be performed at term of pregnancy, though only in selected, quiet, bitches. In 2021, Tal et al. [5] demonstrated the possibility to easily and safely perform amniocentesis in anesthetized bitches at around 35–62 days of pregnancy. Moreover, at the time of parturition, when caesarean section is performed, fetal fluids (amniotic and allantoic) can be easily and securely sampled, as demonstrated by many studies [8,9,10,11,12,13,14,15,16,17,18]. Other than that, the canine fetal fluids were also useful for mesenchymal stem cells isolation and growth as a model of these cell’s biology study and for possible therapeutic application [19,20]. From a hormonal standpoint, canine fetal fluids collected along pregnancy during ovariohysterectomy have been studied also to possibly assess the beginning of thyroid gland function in canine fetuses through the measurement of thyroid hormones [21] and for a better understanding of the fetal endocrine system development [22]. Among the biochemical composition of canine amniotic and allantoic fluids, some studies [10,14] have suggested that amniotic glucose concentrations were associated to the puppies’ outcome. One study [14] reported the finding of a higher concentration of glucose in surviving than non-surviving puppies, demonstrating the relevance of measuring glucose in amniotic fluid to identify newborns with a higher risk of demise after parturition. Moreover, a study [15] highlighted some similarities and differences between the two fetal fluids, pointing out the influence of gender, breed body size and parity on some fetal fluid parameters. In another study [13], fetal fluid concentration of glucose at term was reported to be determined by the maternal serum glucose concentrations.

Despite the undoubted utility of those studies, the traditional timing of laboratories for the analysis of fetal fluids does not fit with the need of a quick and easy tool for the first-line analysis of fetal fluids. In this case, as explained above, the immediate assessment of amniotic glucose concentrations at birth could be helpful for the prompt management of newborns. Different to humans, who give birth at hospitals, where laboratories can immediately process and analyze the biological samples, a dog’s whelping occurs more frequently at the owner’s house or at the breeder’s facility. Even when caesarean section is performed, the prompt analysis of biological samples by a laboratory could be difficult to assure.

In human medicine, the development of the “dry chemistry diagnostic test” allowed the rapid analysis of urines and other biological specimens under every condition, directly by the patient themself, for a quick measurement of some parameters [23]. The “dry chemistry diagnostic test” has highly improved in quality in the last years, providing an increasing assay reliability applicable on multiple biological fluids [23]. Urine test strips are indeed recognized as a first-line diagnostic instrument, characterized by being easy-to-use and able to provide quick, reliable information about the urine composition under several pathological conditions. In humans, urine glucose dipstick tests have also been used to identify the cerebrospinal fluid [24] to underline the usefulness of this tool for the investigation of biological fluids different from urines.

In veterinary medicine, the availability of an easy tool for the immediate evaluation of biological fluid composition on site is even more desirable and beneficial as a first-line diagnostic instrument in many instances and in several animal species. Although marketed for human urine analysis, some studies proved the usefulness of urine analysis reagent strips for the on-field evaluation of uterine lavage fluids in cows [25], for the analysis of urines in the sow [26], and for the evaluation of fetal fluids and urines in newborn dogs [16]. Some authors [11] reported the reliability of semi-quantitative test strip analysis for the on-site differentiation between maternal urines and fetal fluids and defined a first biochemical composition of fetal fluids collected by only one puppy per litter on a total of 23 newborns, highlighting the need for a further evaluation of possible similarities or differences within and among litters.

Based on the above reported reasons, the aim of this study was to assess, by use of commercial semi-quantitative test strips marketed for human urine analysis: (1) possible differences in fluid composition between amniotic and allantoic sacs in surviving and non-surviving newborns born at term of pregnancy from healthy dogs; (2) possible differences in composition between the two types of fluid in surviving puppies; (3) to assess possible effects of maternal age, parity, by newborn gender, Apgar score, and by breed body-size on the composition of fluids in amniotic and allantoic sacs in surviving puppies.

## 2. Materials and Methods

### 2.1. Ethics

The study was performed in agreement with the animal welfare committee ethical guidelines and it was approved by the Ethical Committee of the University of Milan (OPBA_121_2021). All owners, before every procedure of caesarean section, signed a written informed consent, allowing for both surgery and the specific collection of fetal fluids and clinical records for scientific research.

### 2.2. Bitches’ Clinical Management and Fetal Fluid Collection

The study enrolled 37 purebred dogs, 2–9 years old, 1–6 parity, belonging to several breeds. According to bodyweight, the dogs were grouped in small-sized breeds (≤10 kg, *n* = 26; 9 Chihuahua, 4 Maltese, 4 Dachshund, 3 toy Poodle, 3 Jack Russell terrier, 3 Pug) and in a combined class of medium and large-sized breeds (11–40 kg, *n* = 11; 3 Bernese Mountain dog, 2 Leonberger, 2 English Bulldog, 1 Maremma shepherd, 1 Boxer, 1 Hovawart, 1 German shepherd).

Based on individual anamnesis of problems at previous parturition or on the breed-related higher risk for dystocia, elective caesarean sections were performed in all cases for the mothers’ and puppies’ wellbeing. All the dogs were fully monitored from mating and during pregnancy until the time of birth. All dogs were healthy, with a body condition score from 2.5/5 to 3/5. Pregnancy diagnosis and monitoring, and the assessment of fetal development and well-being, were performed as previously reported [8]. The date of parturition was calculated by ultrasonography and elective caesarean section scheduled according to ultrasonographic findings, coupled with the detection of maternal plasma progesterone concentrations ≤2 ng/mL [8]. The anesthetic protocol used [8], which aimed to minimize possible negative effects on newborns, was the same for all the bitches. Without interfering with newborn puppies’ care and resuscitation, after the fetal membranes’ opening, the amniotic and allantoic fluid samples were separate from each fetus and aseptically collected through sterile syringe without a needle into sterile, plain, 10 mL-glass vials [8], avoiding any possible damage to the newborn. Fetal fluid collection, guaranteed by a specific operator, never interfered with the immediate neonatal care and resuscitation, performed by veterinarians specialized in neonatal assistance [8].

### 2.3. Newborn Clinical Evaluation

Soon after parturition, all newborns were evaluated through an APGAR score, and viability classified as previously reported [27]. Moreover, the absence of gross physical malformations was verified and all of the newborns were weighted before nursing. The newborn gender was also recorded. Puppies born alive, with an Apgar score ≥7 at the measurement recorded within 5 min after birth, without gross physical malformations and a normal weight for the related breed, were classified as normal newborns. Low viable (Apgar score < 7), underweighted puppies with gross malformations or fresh born dead puppies were considered as pathologic newborn. Pathologic puppies underwent a different level of neonatal support/resuscitation or euthanasia. At 24 h and at a week after parturition, the puppies’ survival was verified.

### 2.4. Amniotic and Allantoic Fluids Analysis

Contemporary to the immediate newborn clinical evaluation, and always within 5 min after birth, the fetal fluids belonging to each newborn were immediately evaluated by commercial urine test strips (Combur-Test^®^ strips, Roche Diagnostics GmbH, Mannheim, Germany), providing information about the following 10 parameters: specific gravity (SG), pH (pH), leucocytes (L), nitrite (N), glucose (G), protein (P), ketones (K), urobilinogen (U), bilirubin (BR), and erythrocytes (ERY)/hemoglobin (HB). According to the manufacturer instructions, each strip was dipped into the vials containing the amniotic or the allantoic fluid. After the collection and during this procedure, the vials were constantly put on a shaker in order to maintain the homogeneity of the fluid collected and, after dripping on blotting paper, a wait of 60 s was sufficient to have all parameters apart from leucocytes (readable at 120 s). Results for each parameter were recorded in several categories, according to the manufacturer label: specific gravity (Kg/L), ranging from 1000 to 1030; pH, ranging from 5 to 9; leucocytes, ranging from negative to 1 + (~10–25 cells/μL), 2 + (~75 cells/μL), 3 + (~500 cells/μL); nitrites, ranging between negative and positive (0.05 mg/dL); glucose, ranging from negative to 1 + (50 mg/dL), 2 + (100 mg/dL), 3 + (300 mg/dL), 4 + (1000 mg/dL); proteins, ranging from negative to 1 + (30 mg/dL), 2 + (100 mg/dL), 3 + (500 mg/dL); ketons, ranging from negative to 1 + (10 mg/dL), 2 + (50 mg/dL), 3 + (150 mg/dL); urobilinogen, ranging from normal to 1 + (1 mg/dL), 2 + (4 mg/dL), 3 + (8 mg/dL), 4 + (12 mg/dL); bilirubin, ranging from negative to 1 + (0.5 mg/dL), 2 + (not specified), 3 + (6 mg/dL); erythrocyte, ranging from negative to 1 + (~5–10 ery/μL), 2 + (~25 ery/μL), 3 + (~50 ery/μL), 4 + (~250 ery/μL); hemoglobin, ranging from negative to 1 + (~10 ery/μL), 2 + (~25 ery/μL), 3 + (~50 ery/μL), 4 + (~250 ery/μL). Data about erythrocytes/hemoglobin are expressed as “blood”. The sample positivity was considered as the number and percentage of positivity on the total of strips analyzed for each parameter within each group.

### 2.5. Statistical Analysis

The Levene’s t-test was used to evaluate any difference between surviving and non-surviving puppies in both amniotic and allantoic sacs fluids, between amniotic and allantoic fluids inside the group of surviving newborns, and between amniotic and allantoic fluids in small and medium/large-sized surviving puppies. Levene’s test was used for homoscedasticity and the Shapiro–Wilk test for data normality (significance *p* ≤ 0.05). The ANCOVA test was used to assess the possible effect of maternal age and parity, newborn gender, and Apgar score on each parameter in both amniotic and allantoic fluids. Statistical analyses were performed by SPSS 15.0 (SPSS Inc., Chicago, IL, USA) and significance was set at *p* < 0.05.

## 3. Results

### 3.1. Clinical Results

From the 37 dogs with normal pregnancy clinical course, a total of 137 puppies were delivered by elective caesarean section, with a mean ± SD litter size of 4.7 ± 2.18 (range: 1–8); 6.4 ± 1.85 in medium-large and 3.4 ± 1.39 in small-sized breeds. At birth, 127/137 (92.7%) neonates were alive, healthy, and viable (Apgar score ≥ 7) [27], without physical defects, with normal birthweight, and considered as normal newborns. Ten (7.3%) newborns were pathologic: one was stillborn and the other nine were less viable (average Apgar score = 3) and did not respond to the neonatal resuscitation. All the normal puppies alive at 24 h and 7 days after birth were included in the surviving group, while all the pathologic were grouped as non-surviving newborns. Among the surviving newborns, according to maternal breed body-size, 67 (52.8%) puppies belonged to the small-sized breeds and 60 (47.2%) to a merged class of medium–large-sized breeds. In relation to newborn gender, 65 (51.2%) puppies were females and 62 (48.8%) were males.

### 3.2. Amniotic and Allantoic Fluids Analyses

From the surviving newborns, a total of 113 and 107 collections were performed from amniotic and allantoic fluids respectively, while the collection of eight amniotic and eight allantoic fluids was performed on non-surviving newborns.

In some cases, during the collection of specimens, surgical manipulations prevented the collection, or the presence of gross contaminations induced the operator to exclude that sample from the study.

Data about the mean ± SD (range) and number and percentage of the samples’ positivity (italic) for each strip parameter in the amniotic and allantoic fluids of surviving and non-surviving newborns are reported in Table 1.

According to breed body-size, 62 amniotic and 57 allantoic samples were collected from surviving small-sized breeds dogs, while 51 amniotic and 50 allantoic samples were collected in surviving medium/large-sized breeds dogs.

Data about the mean ± SD (range) and the number and percentage of samples’ positivity (italic) for the significant parameters in the 113 amniotic and 107 allantoic canine fluid samples, grouped according to breed body-size, are reported in Table 2.

Statistical analysis did not detect any significant effects played by newborn gender, maternal age, parity, and by Apgar score on all the parameters investigated in both amniotic and allantoic fluids within the group of surviving newborns.

## 4. Discussion

The present study was proposed to assess the usefulness of the on-site evaluation of fetal fluids collected from surviving and non-surviving puppies born at term of pregnancy from healthy dogs by reagent test strip marketed for urine analysis in humans as a possible complementary tool in the clinical evaluation of newborns immediately after birth to allow a more focused neonatal assistance. The first-line and irreplaceable method to assess the viability of puppies is their clinical evaluation through the APGAR score, but the evaluation of fetal fluids may add important information. All the puppies were born by elective caesarean section, and this represents an advantage because of the homogeneity among the puppies’ conditions, but also a weakness because fluids collected by puppies born by emergency caesarean section or vaginal delivery could be different. Moreover, when vaginal delivery occurs, the collection of fetal fluids for analyses could be difficult, limiting the usefulness of this diagnostic tool. Lastly, it should be underlined that an additional person is needed for fluid collection and prompt analysis.

Although a real validation of their use for fetal fluid evaluation is lacking, the commercial reagent test strips have been previously used and validated for uterine lavage fluid evaluation in cows [25], used for the urinalysis in newborn dogs [16], and preliminary results about the use of the marketed urine analysis test strip for fetal fluids evaluation in dogs [11] were reported.

In agreement with the first aim of this study, differences about the ten parameters tested in both fluids (amniotic and allantoic) between surviving and non-surviving newborns were provided. Despite the small number of non-surviving puppies, results showed significantly lower glucose concentrations in the amniotic fluids of non-surviving puppies than surviving, normal subjects. This finding agrees with previous studies, in which lower fetal fluid glucose concentrations were associated with a negative outcome of the puppies [10,14], highlighting the importance of energetic availability for the newborn survival. Because of the importance of providing glucose support as soon as possible to hypoglycemic newborns, this result entails practical advantages. In fact, although it should be considered cautiously, this finding seems to suggest that, under practical settings, the commercial reagent test strips could represent a useful tool, complementary to the newborn clinical examination immediately after birth, allowing a more addressed neonatal assistance. When the mean glucose concentration is considered, the results obtained with the test strips are higher than the 25–30 mg/dL reported in the bibliography [14,15] but in agreement with the higher values reported by Balogh et al. [13]. Therefore, it could be supposed that the test strips may overestimate the actual fetal fluids’ glucose concentrations. The glucose ranges (Negative-100 mg/dL) were similar in most cases apart from allantoic fluid in surviving puppies, in which all the positive reactions accounted to 50 mg/dL. The percentage of positive reaction was high (>70%) in both fluids in surviving and non-surviving newborns, in agreement with the 57–74% positivity reported [11]. The significantly higher amniotic protein concentration in non-surviving than surviving puppies seems to suggest a less efficient protein metabolism in puppies with a negative outcome, or, on the other hand, an increased secretion from fetal compartments. Between the two groups, ranges of protein concentrations were similar in amniotic fluids, while, in the surviving newborns, the allantoic ranges were lower in the minimum value in comparison to non-surviving puppies, evidencing that allantoic protein concentration could result as absent in normal puppies. A positive reaction to proteins was very high (>90%) in both fluids of surviving and non-surviving newborns, in agreement with data obtained with the same test on a small group of normal puppies [11]. The source of the fetal fluid proteins could be the kidneys, but also other organs, such as the ones of the respiratory tract. Proteinuria has been described as a normal finding in newborn puppies [28] due to kidney immaturity. However, [16] reported that, in the urines of 1-day-old puppies, only 8% of puppies showed a positive reaction to proteins (30 mg/dL), suggesting that proteinuria could be detected in a small part of normal newborns. Given that multiple sources contribute to the composition of amniotic fluid, it is difficult to attribute this finding solely to a renal lower efficiency, but instead, a multi-system efficiency impairment could be hypothesized. Moreover, it has been suggested that allantoic fluid could more likely reflect the fetal urines, even if they also concur in the amniotic fluid’s composition [15]. Therefore, the absence of significant differences between surviving and non-surviving allantoic protein concentrations seems to suggest that not only the renal function should be considered in the possible explanation of this result. However, the finding of significantly higher concentrations of both amniotic and allantoic nitrites (and of the percentage of positive reactions) in non-surviving than surviving puppies seems to corroborate the hypothesis of a less efficient system of detoxification in newborns with a negative outcome. It should be noted that, although a small number of non-surviving newborns, 25% of subjects showed a positive reaction to nitrites in both amniotic and allantoic fluids in this group in comparison to the 3–0% (amniotic and allantoic fluids, respectively) in the surviving group. Concerning the recorded intervals, nitrites’ ranges were similar, except for allantoic fluid in surviving puppies, which always resulted as negative. The higher concentrations of allantoic urobilinogen in non-surviving newborns could indicate an impaired function in the hepatic or renal function, with consequent excretion in fetal urines. Despite the similar ranges, the percentage of positive reaction was notably higher in non-surviving than surviving puppies (13% vs. 5%). The finding of higher amniotic bilirubin concentrations in non-surviving than surviving newborns could be attributable to a hepatic and/or renal impaired function (or immaturity). Moreover, the percentage of positive reaction was higher in non-surviving than surviving puppies (25% vs. 4%), and the bilirubin ranges were higher in non-surviving than surviving amniotic fluids, highlighting once more the difference between the two groups of puppies.

When surviving puppies are concerned, the results from the present study evidenced some differences between the amniotic and the allantoic fluids, in accordance to literature [11,13,15] both in dogs and [6] in cats, probably reflecting the specificity of the canine amniotic and allantoic sacs. Although the amniotic sac has a scarce vascularization, blood vessels show a parallel route between the amniotic cells and the allantoic membrane, allowing the diffusion from amniotic cells to allantoic vessels [29], possibly explaining the similarities of the amniotic and allantoic fluids with regards to biochemical aspects. However, the differences found could probably reflect the peculiar metabolism and transport function, coupled with the specific contribution from the fetal and placental parts to the allantoic and amniotic compartments [30]. In the present study, a significantly higher mean specific gravity, higher bilirubin concentrations, and a significantly lower mean pH was measured in allantoic in comparison to amniotic fluids. The higher specific gravity in allantoic than amniotic fluids was also reported [11] in a small number of puppies using a similar test strip. The 1005–1025 Kg/L range found in the present study for both fluids also agree with the 1005–1022 Kg/L previously reported [11] using the same test strip, though was slightly different from the 1002-1016 reported by Tal et al. [5] in canine fetal fluids collected at 35–62 days of pregnancy. A lower pH in allantoic than amniotic fluid was also found by [11], and could be related to the higher major contribution of fetal urines in the allantoic than in the amniotic compartment at the end of pregnancy. It was indeed reported [16] that, in 1-day-old normal puppies, the urinary pH measures 5.3 ± 0.6. The range of pH was identical to the one reported [11] for allantoic fluid, and was similar for amniotic fluid. The higher bilirubin concentrations in allantoic than amniotic fluids were also found in a previous study [15]. The diverse ranges observed in the two fluids, and the different percentage of positive reactions (4 vs. 40%), agree with data previously reported [11]. According to the suggestion of some authors [11], the finding of higher bilirubin concentrations in allantoic than amniotic fluids could be attributable to the possible excretion of bilirubin within the fetal urines. However, one of the sources could be placental marginal hematoma, from which the uteroverdin is transformed in bilirubin. At last, differently from a previous work [11], no significant differences in blood concentrations were detected between amniotic and allantoic fluids, and no differences between the two fluids were found for protein concentrations, unlike data reported by [15].

In a dog, large differences exist among the many recognized breeds, mainly related to the different body sizes. A study [8] evidenced different IGF-I concentrations in amniotic and allantoic fluids collected from small and medium–large breeds. In two studies [13,15], allantoic glucose levels were higher in medium/large-sized than in small-sized breeds. More recently, a higher leptin amniotic concentration was found in small-sized than large-sized canine breeds [18], evidencing once more a different metabolism in fetuses from small-sized and large-sized breeds, measurable in fetal fluids. In the present study, significantly higher glucose levels were found in the amniotic and allantoic sacs of medium/large breeds when compared to small-sized breeds. This result is partially in agreement with previous data by [5,13], in which higher glucose concentrations in medium/large-sized than small-sized dogs were found only in the allantoic fluids. The ranges of glucose concentrations and percentages of positive reactions were similar in both fluids and in both small and medium/large-sized breeds. The different fetal fluids’ glucose concentrations independent of the breed size could be explained by a diverse metabolism, related to different body size or even to different canine breeds. In the present work, a significantly higher protein content was found in both fluids in small than medium-large-sized breeds, corroborating the previous hypothesis of a different metabolism in relation to breed size. However, because of the marked differences between canine breeds belonging to the same body-size, and because breed-related metabolic differences could be a possibility, further studies focusing on specific breeds should be expected. Therefore, the breed-size differences found in the present study should be considered cautiously, also because of the relatively high number of different breeds within each group. The finding of the same difference in both amniotic and allantoic fluids seems to suggest that, beside the possible effect played by the mother, the fetal protein metabolism also contributes to the different fetal fluids’ final composition. More difficult to explain is the different amniotic and allantoic pH, higher in medium-large than in small-sized breeds. In fact, although a possible effect played by the different litter-size breeds could be supposed, at least for the amniotic pH, it should be expected to find an opposite result, with higher pH in the smallest litters. Therefore, a different explanation should be considered, implying, once again, a different metabolism between small and medium/large-sized breeds. If fetal fluids pH could reflect the blood pH of the fetus, it must be underlined that, in the present study, all the enrolled puppies were normal and viable with an Apgar score ≥7 [27] independent of the breed-size. Even if it was reported [31] that all the puppies experienced a short phase of acidemia immediately after birth, it remains difficult to speculate whether or not these blood changes could occur immediately before birth, leading to a significant different contribution to the final pH in amniotic than allantoic fluid. The finding of lower amniotic and allantoic bilirubin concentrations in small than medium/large-sized dogs with higher percentages of positive reactions in allantoic samples seems to suggest, once more, that the fetal metabolism at term of pregnancy, with the hepatic and renal function and bilirubin excretion, could be different between dogs of different body-sizes and, maybe, breeds. In relation to the percentages of positive reactions, no notable differences were observed for most parameters. However, the pH ranges showed a lower minimum value for amniotic fluid in small-sized puppies and a higher maximum value in allantoic fluid in medium/large-sized puppies.

No significant differences were found between both fetal fluids’ composition in relation to newborn gender. These findings contrast, for example, with the finding of higher glucose concentrations in the amniotic fluids of males when compared to females for normal puppies [14], but agrees with the absence of significant differences in glucose amniotic concentrations [15]. No significant differences were detected by statistical analysis in the fetal fluids’ composition of surviving puppies in relation to the Apgar score, in agreement with previous results [15]. In this study, moreover, no significant effect of maternal age and parity was found for all the studied parameters. This finding disagrees with the negative correlation previously reported [15] for amniotic and allantoic glucose concentrations and maternal parity. Therefore, a better understanding of the actual influence of maternal age and parity on fetal fluids’ composition needs further investigation.

A last mention to the percentages of positive reactions and ranges for those parameters that did not show significant differences at the statistical analysis. In the present study, leukocytes showed higher percentages of positive reactions in both fluids, slightly higher in surviving than non-surviving newborns, with ranges from negative to 75 cells/μL. The percentage of positivity to leukocytes (0–17%) was, however, slightly lower than the about 50% reported by [11]. Ketones’ positive reactions were also higher in non-surviving than surviving newborns in both fluids, but with similar ranges between negative and 10 mg/dL. Blood positive reaction was always very high (>80%), with ranges between negative and 250 ery/μL. The high percentages of positive reaction to blood, and maybe to leukocytes, in both fetal fluids could be explained by the characteristic of the dog placenta to present the marginal hematoma of the placental girdle, where the blood was suggested to be useful as an iron source for the fetus [29]; however, in agreement with [11], the possible contamination with blood at the time of collection could also have caused the high positive reaction in both fluids. In this regard, it should be noted that Tal et al. [5] reported only 5.8% of placental small hematomas occurring during amniocentesis in anesthetized dogs, performed by different techniques at 35–62 days of pregnancy.

## 5. Conclusions

The findings of this study demonstrated the usefulness of commercial reagent test strips for urine analysis for the quick evaluation of fetal fluids in puppies at elective caesarean section. Although the small number of non-surviving newborns, the differences between surviving and non-surviving puppies seem to suggest that the on-site analysis of fetal fluids’ composition by reagent test strips could represent a first-line tool for the evaluation of puppies and for the quick identification of newborns needing special monitoring or assistance. The comparison between the obtained results and data provided in the bibliography by traditional laboratory analyses cautiously suggest the reliability of the reagent test strips for the on-field assessment of fetal fluids’ composition as a complementary tool in the clinical evaluation of the newborn dog. Further studies should clarify the effect of emergency caesarean section or vaginal delivery on fetal fluids’ composition assessment by reagent test strips. For some parameters, however, further investigations are needed to better define their significance in the clinical assessment of newborn dogs.

## Figures and Tables

**Table 1:** Mean ± SD (range) and number and percentage of samples’ positivity (italic) for each strip parameter in amniotic and allantoic fluids of surviving and non-surviving newborns.

	Surviving Newborns (*n* = 127)		Non-Surviving Newborns (*n* = 10)	
	Amniotic Fluid (*n* = 113)	Allantoid Fluid (*n* = 107)	Amniotic Fluid (*n* = 8)	Allantoic Fluid (*n* = 8)
Specific gravity (Kg/L) add bottom border	1010 ± 6.50 ^a^ (1005–1025)	1013 ± 7.14 ^b^ (1005–1025)	1009 ± 8.76 (1005–1030)	1009 ± 5.85 (1005–1020)
pH	7.2 ± 0.78 ^a^ (6–8)	6.5 ± 0.99 ^b^ (5–8)	6.8 ± 1.28 (5–8)	6.8 ± 0.99 (5–8)
Leucocytes (cells/μL)	5.6 ± 14.60 (Negative–75) *19 (17%)*	7.1 ± 18.50 (Negative–75) *18 (17%)*	0.0 ± 0.00 (Negative–Negative) *0 (0%)*	3.1 ± 8.84 (Negative–25) *1 (12.5%)*
Nitrites (mg/dL)	0.0 ± 0.01 * (Negative–0.05) *3 (3%)*	0.0 ± 0.0 * (Negative–Negative) *0 (0%)*	0.01 ± 0.02 * (Negative–0.05) *2 (25%)*	0.02 ± 0.03 * (Negative–0.05) *2 (25%)*
Glucose (mg/dL)	81.3 ± 28.40 * (Negative–100) *82 (73%)*	50.0 ± 0.00 (50–50) *81 (76%)*	50.0 ± 26.73 * (Negative–100) *6 (75%)*	46.7 ± 27 (Negative–100) *7 (88%)*
Proteins (mg/dL)	134 ± 149 * (30–500) *113 (100%)*	130 ± 149 (0–500) *100 (94%)*	333 ± 232 * (30–500) *8 (100%)*	233 ± 207 (100–500) *8 (100%)*
Ketons (mg/dL)	0.6 ± 2.44 (Negative–10) *7 (6%)*	0.6 ± 2.32 (Negative–10) *6 (6%)*	1.3 ± 3.54 (Negative–10) *1 (13%)*	1.7 ± 4.08 (Negative–10) *1 (13%)*
Urobilinogen (mg/dL)	0.01 ± 0.09 (Negative–1) *1 (1%)*	0.1 ± 0.43 * (Negative–4) *5 (5%)*	0.0 ± 0.00 (Negative–Negative) *0*	0.7 ± 1.63 * (Negative–4) *1 (13%)*
Bilirubin (mg/dL)	0.02 ± 0.10 ^a,^* (Negative–0.5) *5 (4%)*	0.6 ± 1.24 ^b^ (Negative–6) *43 (40%)*	0.4 ± 1.05 * (Negative–3) *2 (25%)*	1.2 ± 1.44 (Negative–3) *4 (50%)*
Blood (Ery/μL)	73.7 ± 87.70 (Negative–250) *93 (82%)*	88.1 ± 96.90 (Negative–250) *89 (83%)*	70.0 ± 74.10 (10–250) *8 (100%)*	65.8 ± 92.00 (10–250) *8 (100%)*

^a,b^ within row denotes significant differences with *p* < 0.05 between amniotic and allantoic fluid in normal puppies; * within row denotes significant differences with *p* < 0.05 between amniotic and allantoid fluid in normal and pathologic newborns.

**Table 2:** Data about the mean ± SD (range) and the number and percentage of samples’ positivity (italic) for the significant parameters in the 113 amniotic and 107 allantoic canine fluid samples collected from surviving puppies, grouped according to breed body-size.

	Amniotic Fluid		Allantoic Fluid	
	Small-Sized (*n* = 62)	Medium/Large-Sized (*n* = 51)	Small-Sized (*n* = 57)	Medium/Large-Sized (*n* = 50)
pH	7.1 ± 0.86 ^a^ (5–9)	7.4 ± 0.65 ^b^ (6–8)	6.2 ± 0.91 ^A^ (5–8)	6.7 ± 1.02 ^B^ (5–9)
Glucose (mg/dL)	49 ± 29.57 ^a^ (0–100) *50 (80%)*	56 ± 19.27 ^b^ (0–100) *49 (96%)*	44 ± 29.98 ^A^ (0–100) *43 (75%)*	50 ± 22.82 ^B^ (0–100) *44 (88%)*
Proteins (g/L)	194.9 ± 176.31 ^a^ (30–500) *62 (100%)*	59.4 ± 34.90 ^b^ (30–100) *51 (100%)*	165.6 ± 167.11 ^A^ (negative–500) *56 (98%)*	89.4 ± 113.00 ^B^ (negative–500) *44 (88%)*
Bilirubin (mg/L)	0.02 ± 0.09 ^a^ (negative–0.5) *2 (3%)*	0.03 ± 0.12 ^b^ (negative–0.5) *3 (6%)*	0.4 ± 0.84 ^A^ (negative–0.5) *22 (39%)*	0.9 ± 1.55 ^B^ (negative–0.5) *21 (42%)*

^a,b^ and ^A,B^ within row denote significance with *p* < 0.05 between amniotic and allantoic fluids in small and medium/large sized breeds.

## Data Availability

The data presented in this study are available upon request to the corresponding author.

## References

[1] Underwood M.A., Gilbert W.M., Sherman M.P. (2005). Amniotic fluid: Not just fetal urine anymore. J. Perinatol..

[2] Cruz-Lemini M., Parra-Saavedra M., Borobio V., Bennasar M., Goncé A., Martínez J.M. (2014). How to perform an amniocentesis. Ultrasound Obstet. Gynecol..

[3] Musilova I., Bestvina T., Stranik J., Stepan M., Jacobsson B., Kacerovsky M. (2017). Transabdominal amniocentesis is a feasible and safe procedure in preterm prelabor rupture of membranes. Fetal Diagn. Ther..

[4] Chavatte-Palmer P., Tarrade A. (2016). Placentation in different mammalian species. Ann. Endocrinol..

[5] Tal S., Kahila Bar-Gal G., Arlt S.P. (2021). Evaluation of short-termsafety of ultrasound-guided foetal fluid sampling in the dog (*Canis lupus familiaris*). Vet. Rec.

[6] Fresno L., Rodriguez-Gil J.E., Rigau T., Pastor J., Rivera del Alamo M.M. (2012). Modulation of the biochemical composition of amniotic and allantoic fluids as a control mechanism of feline foetal development. Placenta.

[7] Bonte T., Del Carro A., Paquette J., Charlot Valdieu A., Buff S., Rosset E. (2017). Foetal pulmonary maturity in dogs: Estimated from bubble tests in amniotic fluid obtained via amniocentesis. Reprod. Domest. Anim..

[8] Meloni T., Comin A., Rota A., Peric T., Contri A., Veronesi M.C. (2014). IGF-I and NEFA concentrations in fetal fluids of term pregnancy dogs. Theriogenology.

[9] Dall’Ara P., Meloni T., Rota A., Servida F., Filipe J., Veronesi M.C. (2015). Immunoglobulins G and lysozyme concentrations in canine fetal fluids at term of pregnancy. Theriogenology.

[10] Groppetti D., Martino P.A., Ravasio G., Bronzo V., Pecile A. (2015). Prognostic potential of amniotic fluid analysis at birth on canine neonatal outcomes. Vet. J..

[11] Balogh O., Roch M., Keller S., Michel E., Reichler I.M. (2017). The use of semi-quantitative tests at Cesarean section delivery for the differentiation of canine fetal fluids from maternal urine on the basis of biochemical characteristics. Theriogenology.

[12] Bolis B., Prandi A., Rota A., Faustini M., Veronesi M.C. (2017). Cortisol fetal fluid concentrations in term pregnancy of small-sized purebred dogs and its preliminary relation to first 24 hours survival of newborns. Theriogenology.

[13] Balogh O., Bruckmaierb R., Keller S., Reichlera I.M. (2018). Effect of maternal metabolism on fetal supply: Glucose, nonesterified fatty acids and beta-hydroxybutyrate concentrations in canine maternal serum and fetal fluids at term pregnancy. Anim. Reprod. Sci..

[14] Bolis B., Scarpa P., Rota A., Vitiello T., Veronesi M.C. (2018). Association of amniotic uric acid, glucose, lactate and creatinine concentrations and lactate/creatinine ratio with newborn survival in small-sized dogs—preliminary results. Acta Vet. Hung..

[15] Veronesi M.C., Bolis B., Faustini M., Rota A., Mollo A. (2018). Biochemical composition of fetal fluids in at term, normal developed, healthy, viable dogs and preliminary data from pathologic littermates. Theriogenology.

[16] Melandri M., Veronesi M.C., Alonge S. (2020). Urinalysis in Great Dane Puppies from Birth to 28 Days of Age. Animals.

[17] Miller I., Schlosser S., Palazzolo L., Veronesi M.C., Eberini I., Gianazza E. (2020). Some more about dogs: Proteomics of neglected biological fluids. J. Proteom..

[18] Veronesi M.C., Fusi J., Comin A., Ferrario P.G., Bolis B., Prandi A. (2020). Effect of breed body-size on leptin amniotic fluid concentrations at term pregnancy in dogs. Theriogenology.

[19] Filioli Uranio M., Valentini L., Lange-Consiglio A., Caira M., Guaricci A.C., L’Abbate A., Catacchio C.R., Ventura M., Cremonesi F., Dell’Aquila M.E. (2011). Isolation, Proliferation, Cytogenetic, and Molecular Characterization and in Vitro Differentiation Potency of Canine Stem Cells from Foetal Adnexa: A Comparative Study of Amniotic Fluid, Amnion, and Umbilical Cord Matrix. Mol. Reprod. Dev..

[20] Iacono E., Marcoccia R., Merlo B. (2021). Current Status on Canine Foetal Fluid and Adnexa Derived Mesenchymal Stem Cells. Animals.

[21] Thuróczy J., Szilágyi J., Müller L., Balogh L. (2017). Development of the independent function of fetal thyroid glands in the dog in connection with iodothyronine concentrations in pregnant bitches, fetal fluids, and fetal serum. Domest. Anim. Endocrinol..

[22] Thuróczy J. (2020). Foetal development of endocrine organs in dog. Reprod. Domest. Anim..

[23] Gupta A., Dwivedi T. (2019). Reagent strips test: A semplified method for prompt analysis of cerebrospinal fluid in neurological disorders in emergency. Pract. Lab. Med..

[24] Hamilton R., Young S. (2007). Identifying CSF using urine glucose dipstick testing. Int. J. Obstet. Anesth..

[25] Ricci A., Bonizzi G., Sarasso G., Gallo S., Dondo A., Zoppi S., Vincenti L. (2017). Subclinical endometritis in beef cattle in early and late postpartum: Cytology, bacteriology, haptoglobin and test strip efficiency to evaluate the evolution of the disease. Theriogenology.

[26] Mazutti K., Locatelli-Dittrich R., Lunardon I., Kuchiishi S.S., de Lara A.C., Zotti E., Alberton G.C. (2013). Evaluation of the reagent test strips and microscopic examination of urine in the diagnosis of urinary tract infection in sows. Pesq. Vet. Bras..

[27] Veronesi M.C., Panzani S., Faustini M., Rota A. (2009). An Apgar scoring system for routine assessment of newborn puppy viability and short-term survival prognosis. Theriogenology.

[28] Peterson M.E., Peterson M.E., Kutzler M.A. (2011). Neonatal mortality. Small Animal Pediatrics.

[29] Miglino M.A., Ambrósio C.E., dos Santos Martins D., Wenceslau C.V., Pfarrer C., Leiser R. (2006). The carnivore pregnancy: The development of the embryo and fetal membranes. Theriogenology.

[30] Li N., Wells D.N., Peterson A.J., Lee R.S. (2005). Perturbations in the biochemical composition of fetal fluids are apparent in surviving bovine somatic cell nuclear transfer pregnancies in the first half of gestation. Biol. Reprod..

[31] Lúcio C.F., Silva L.C.G., Rodrigues J.A., Veiga G.A.L., Vannucchi C.I. (2009). Acid-base changes in canine neonates following normal birth or dystocia. Reprod. Domest. Anim..

